# Analysis of Performance Parameters of the Smash in Male and Female Professional Padel

**DOI:** 10.3390/ijerph17197027

**Published:** 2020-09-25

**Authors:** Bernardino J. Sánchez-Alcaraz, Daniel T. Perez-Puche, Francisco Pradas, Jesús Ramón-Llín, Alejandro Sánchez-Pay, Diego Muñoz

**Affiliations:** 1 Department of Physical Activity and Sport, Faculty of Sport Sciences, University of Murcia, C/Argentina, s/n, 30700 Murcia, Spain; bjavier.sanchez@um.es (B.J.S.-A.); danieltomas.perezp@um.es (D.T.P.-P.); 2 Department of Musical, Plastic and Corporal Expression, Faculty of Human Sciences and Education, University of Zaragoza, 50009 Zaragoza, Spain; franprad@unizar.es; 3 Department of Musical, Plastic and Corporal Expression, University of Valencia, 46003 Valencia, Spain; jesus.ramon@uv.es; 4 Department of Musical, Plastic and Corporal Expression, Faculty of Sport Sciences, University of Extremadura, 06006 Extremadura, Spain; diegomun@unex.es

**Keywords:** racket sports, technique, performance analysis, tactics

## Abstract

The aim of this study was to analyze the distribution and effectiveness of the different types of smash in professional padel according to the area and direction of the strokes and the gender. Through systematic observation, 1.015 smashes from eight finals (four men’s and four women’s) of the professional matches were analyzed. The smashes were categorized into four types of smash: tray, flat, topspin and off the wall. The results showed both men’s and women’s that the tray is the most used smash by padel players, presenting a percentage of point continuity of almost 90%. The flat and topspin smashes are the strokes that achieve the highest percentage of winning points (near 60%), although this efficiency decreases significantly when the players move away from the net area (*p* < 0.05), especially in the flat smash. Men perform a higher percentage of winning smashes than women, mainly in the flat smash (*p* = 0.02). Furthermore, with regards to direction, flat and off the wall smashes are predominantly down the line strokes and women perform significantly more cross court topspin smashes than men (*p* = 0.005). The results shown could be used to design tasks and exercises by padel coaches at professional players.

## 1. Introduction

Padel is a racket sport that was born in Mexico approximately 50 years ago [1] and has experienced enormous growth in the last decade both in the number of players and in the facilities for practicing it [2,3]. Currently it is practiced in more than 40 countries and it has an international tournament circuit in which the best padel players in the world participate [4]. This greater professionalization of padel has also produced an increase in scientific publications [5], especially those related to the performance analysis of the sport [6,7]. In this respect, most research has focused on three fundamental aspects: temporal parameters [8,9,10,11], players’ movements and distance covered on the court [12,13,14,15] and game actions [16,17]. The results of these investigations have an enormous transfer and practical application in the design of training sessions adapted to the characteristics of the competition [18,19].

These studies, carried out on professional players, have determined that there are two basic tactical positions in padel: the offensive position, where the players play close to the net and the defensive position, where the players play near the baseline of the court [20]. Previous studies indicate the importance of occupying and maintaining positions close to the net to increase the likelihood of success [17,21]. Some research show that more than 80% of winning points are won from the offensive position and the winning players perform more attack strokes per point and per game [22]. Thus, the most commonly used strokes by players in the offensive position are volleys, followed by trays and smashes [12,17,23,24]. Moreover, 80% of the points in padel are finished with less than three attack shots, which reveal the offensive nature of such hits [22].

There is a continuous dichotomy during the development of the point, where players who are at the net try to keep this advantageous position, while the backcourt players try to recover it [21,25]. Players in the defensive position also perform different types of technical actions such as the lob or passing shot, varying both the height and direction of the strokes, with the aim of displacing the attacking pair so that they hit from more forced positions [7,26]. Therefore, the lobs will cause attacking players to hit the ball going above their heads. Over-head strokes (smash and tray) are the most successful shots during a match along with cross court lobs [12,27]. They are played from the middle and the net area, to maintain a positional advantage and increase the chances of winning a point [16,28]. The success of the smash as a winner depends, amongst other factors, on the area, direction, velocity and accuracy with which is executed [22,29]. The smash has become a very important and decisive shot. No previous studies have focused on this topic, even less observe the differences between males and females, which could affect the design of training sessions, depending on the gender. Due to the anthropometric and strength differences between male and female padel players, our hypotheses is that a male should use a more flat smash than a female to win the point, and the tray should be more used by women. Finally, for male and female players it could be possible that they have different behavior in the stroke direction.

Following the review conducted, there is research related to a performance analysis in padel, but mainly in male players. Research focused only on a female padel player is scarce [7]. In relation to match analysis, rally length or shots per rally are higher in female than male matches [17]. In addition, in the professional category, females perform 4% more smashes than males [17]. It seems therefore that the male and female matches develop differently and knowing the characteristics of one of the most decisive strokes in padel it would help to understand the differences between winning and losing according to gender. Thus, because of the importance of the smash as a decisive stroke in the point, the objective of this study will be to analyze the distribution and effectiveness of the different types of smash in professional padel depending on the area and direction of the shot and the gender of the players.

## 2. Materials and Methods

### 2.1. Sample and Variables

**Figure 1:**
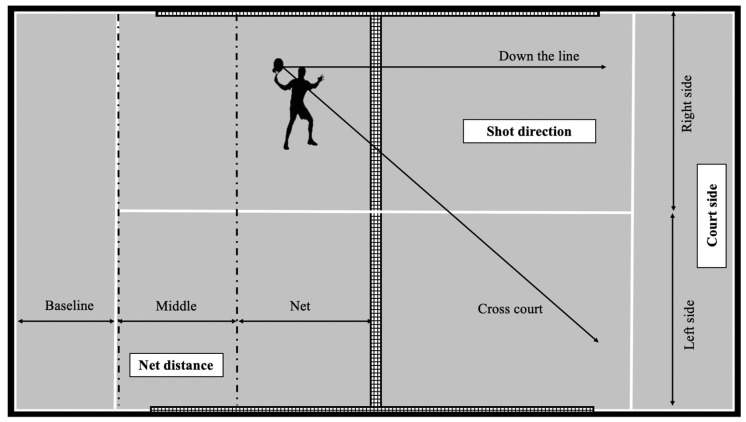
Hitting zones (net distance and court side) and shot direction.

It is a descriptive and observational study of quantitative methodology. The sample included 1.015 smashes corresponding to eight finals (four men’s and four women’s matches) of the official circuit World Padel Tour 2019 held in Barcelona, Valladolid, Madrid, Santander and Murcia (Spain). The analyzed smashes were made by 20 professional padel players: 10 men (age = 32.35 ± 6.28 years old) and 10 women (age = 29.62 ± 5.91 years old) and the study was conducted in accordance with the Declaration of Helsinki of 2013. The following variables were analyzed:

- Type of smash: The different types of smash were classified into four shots, following the classification proposed by other authors in padel research [16,22]:
  1. Tray: Offensive stroke, without a bounce, which is made over the head and on the dominant side of the player. In this shot, before hitting the ball, the player opens the face of the racket pointing upwards and hits with a slice effect. The impact point on the ball is lower than in the other smashes.
  2. Flat smash: Offensive stroke, without a bounce, which is made over the head and on the dominant side of the player. In the execution of this shot, the player hits the ball with a lot of power at the highest possible point, with a flat stroke (no effect), so that after bouncing on the opposite side, the ball could go out of court or return to the other side after rebounding against the wall.
  3. Topspin smash: Offensive stroke, without a bounce, made over the head and hitting the ball from the non-dominant side (behind the player’s head). In the execution of this shot the player hits the ball with a lot of power, with a topspin effect, accelerating the shot by arching the back so that, after bouncing the ball on the wall of the opposite side, it goes out over one of the sides walls of the court.
  4. Off the wall smash: Offensive stroke, with a bounce, which is made above the head and on the dominant side of the player. This shot is made when the player, after receiving a lob, lets the ball bounce on his/her side and waits for the bounce on his/her back wall to make a smash. Depending on the player’s aim, this shot can be done with a flat or slice effect.
- Shot effectiveness: The classification proposed by Courel-Ibáñez and Sánchez-Alcaraz (2017) was used to determinate smash effectiveness, distinguishing between the winner (the attacking player wins the point by making a smash), error (the attacking player fails the smash and loses the point) or continuity (the point continues after the smash).
- Hitting zone: The court was divided into six zones, depending on the court side (right or left) and the distance to the net (net, middle and baseline) when the player hit the ball (Figure 1). Baseline zone, from wall to the serve line (3 m); middle zone, from the serve line to 1/3 court area (3.5 m); net zone, from 1/3 court area to net (3.5 m).
- Shot direction: Two possible trajectories were distinguished: down the line and cross court (Figure 1).

### 2.2. Procedure

Data were collected through systematic observation, carried out by two observers who have a degree in Sports Science and are specialized in padel. Observers were specifically trained in the use of the observational instrument during two weeks. The training focused on the clear identification of the variables (type of smash, shot effectiveness, hitting zone and shot direction) and the use of the observational instrument software. At the end of the training process, each observer analyzed the same two sets in order to calculate the inter-observer reliability through the Multirater Kappa Free [30], obtaining values above 0.80. To ensure the consistency of the data, intra-observer reliability was evaluated at the end of the observation process, obtaining minimum values of 0.89. The kappa values obtained revealed a very high degree of agreement (>0.80) [31]. All the analyzed matches were retransmitted in streaming and later hosted on the World Padel Tour website (https://www.worldpadeltour.com/) [32], from where they were downloaded for the observation, collection and analysis of the data. LINCE specialized software was used for this process of recording and data collection [33].

### 2.3. Data Analysis

Firstly, a descriptive analysis of the data was carried out and the mean (M), standard deviation (SD), frequency (*n*) and percentage (%) were calculated on the whole sample. A comparison was made of the statistics of distribution and efficiency of the smashes according to gender and the playing side of the court using Pearson’s Chi-Square test. Column proportions were compared using Z tests on the effectiveness of the smash according to the area and gender of the players. A significance level of *p* < 0.05 was established, which was adjusted according to Bonferroni in the Z tests. The associations among the categories of the variables were performed with corrected standardized residuals (CSR). The effect size was calculated using Crammer’s V [34]. All data was analyzed with the IBM SPSS 25.0 statistical package for Macintosh (IBM Corp: Armonk, NY, USA).

## 3. Results

**Figure 2:**
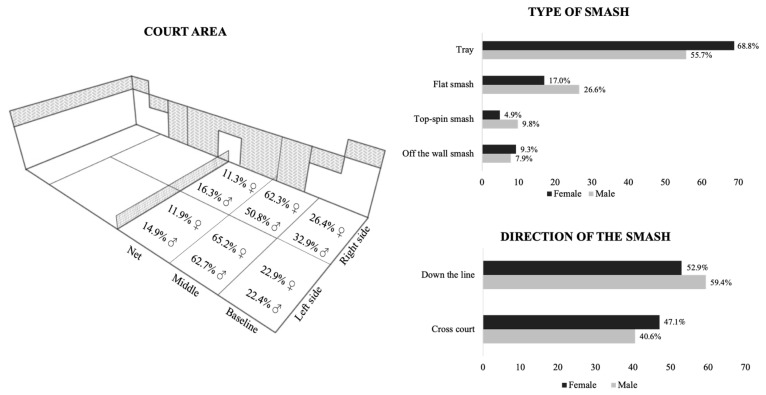
Percentages of distribution of all the smashes according to the area of the court (diagram on the left), type of smash (black bars) and direction of the smash (grey bars) by gender.

Figure 2 shows the results of the distribution percentages of the different smashes analyzed according to the type of smash, direction of hitting and court area comparing by gender. According to smash frequency, significant differences were found between genders (χ^2^ = 26.423; gl = 3; *p* < 0.001; *V* = 0.161). The female player performed significantly more tray strokes than male (*CSR* = 4.3. Otherwise, the male player made more flat (*CSR* = 3.7) and top-spin smashes (*CSR* = 3.0) than female players. Depending on the area of the court, most of the smashes were made in the middle area of the court for both genders. In relation to the direction of the smash, male players performed significantly more down the line strokes than female (χ^2^ = 4.281; gl = 1; *p* = 0.039; CSR = 2.1; *V* = 0.065).

Depending on the hitting side, there was a trend towards a higher percentage of smashes in the middle zone in both genders. On the left side of the court, the smashes made in the different areas of the court were similar between male and female (χ^2^ = 2.371; gl = 2; *p* > 0.05; *V* = 0.065). By contrary, in the right side of the court male and female players played different (χ^2^ = 9.497; gl = 2; *p* < 0.05; *V* = 0.145). Male players made more smashes in the net (*CSR* = 1.8) and in the baseline (*CSR* = 1.5), and females played more smashes in middle area (*CSR* = 2.4).

**Figure 3:**
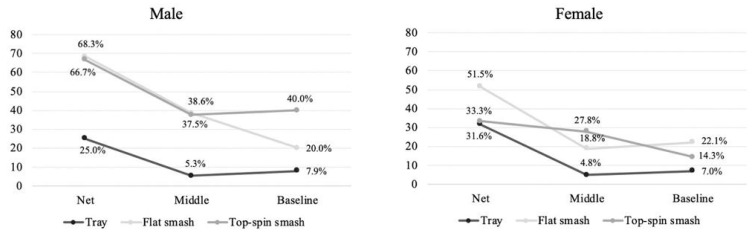
Percentage of winning points depending on the type of smash and the area of the court by gender.

Figure 3 shows the percentage of winning points depending on the type of smash and the area of the court by gender. In general lines, male and female players make more winning smashes in the net area than the others. Moreover, male players made more flat and top-spin smashes than females regardless of the hitting area. On the contrary, the female players made more winner shots with the tray stroke only at the net area.

The division by the type of smash (Table 1) showed differences in the distribution of hitting efficiency according to the gender of the players in the flat smash (χ^2^ = 7.833; gl = 2; *p* < 0.05; *V* = 0.187). The men performed a significantly higher percentage of winning flat smashes (CSR = 2.7) and less percentage of continuity of this stroke than the women (CSR = −2.8). No significant differences were found according to gender for the tray (χ^2^ = 3.332; gl = 2; *p* > 0.05), topspin (χ^2^ = 2.376; gl = 2; *p* > 0.05) and off the wall smashes (χ^2^ = 1.333; gl = 2 *p* > 0.05). Furthermore, the flat smash and topspin smash were the two smashes that produced more winners, while the tray and off the wall smashes were the smashes recording a higher percentage of continuity. The smash with the highest percentage of errors was the off the wall smash.

Table 2 shows the differences in the percentages of distribution of the smash direction regarding players’ gender and type of smash. Players’ gender significantly determined the direction of the topspin smash (χ^2^ = 7.203; gl = 1; *p* < 0.01; *CSR* = 2.7; *V* = 0.31). Thus, while men equally distributed the direction of their topspin smashes, women performed more than 80% of their topspin smashes cross court. No significant differences were found between men and women in the direction of the flat smash (χ^2^ = 2.635; gl = 1; *p* > 0.05), tray (χ^2^ = 0.260; gl = 1; *p* > 0.05) and off the wall smash (χ^2^ = 0.152; gl = 1; *p* > 0.05). In general, both men and women hit a higher percentage of down the line flat and off the wall smashes, while the tray was directed equally down the line and cross court.

## 4. Discussion

Smash stroke is the game actions that produce the highest percentage of winner shots in padel [12,27] and has a great influence in match outcome. For that, the aim of this study was to analyze the distribution and effectiveness of the different types of smashes in professional padel according to court area, smash direction and players’ gender. The results obtained showed that the tray stroke is the most widely used smash type, mainly for female players (Figure 2). In addition, this hit means, in almost 90% of cases, the continuity of the point, which would indicate an important technical domain for the players, since there are hardly any errors [26], although it is also easier for the opponents to defend [17]. On the other hand, the distribution of the smashes by court area showed that approximately 60% of the smashes were made in the middle area. These data confirm that the defending players seek to make high and deep lobs forcing the attacking players to hit the ball in situations far from the net, reducing the possibilities of achieving a winner [16,22]. In addition, on the right-side male players performed a greater number of smashes at the net and baseline areas than female. In the baseline area these differences could be due to anthropometric and strength differences that would allow the male players to make effective smashes further from the net. Regarding the smash effectiveness according to the hitting area (Figure 3), data showed that there is a direct relationship between the distance to the net in the hit and its effectiveness (mainly from the net area with the others). Thus, as the players hit closer to the net, the number of winners increased significantly, especially when using topspin and flat smashes. This could be explained by a favorable position for the attackers (decreasing the possibility of an error), and a shorter reaction time for the defenders. In this sense, coaches should include a high percentage of tray strokes in the training session (with the aim of keeping opponents away from the net), as well as working shots close to the net as a means of finishing the point.

Previous studies in professional padel have shown that players who are in defensive positions predominantly use the lob stroke to recover the net [7,26,35]. However, the attacking players try to maintain the offensive position [22], due to the greater probabilities of winning the point when they are in situations close to the net [16,17,22]. Therefore, in order to maintain the attacking position, it seems that players try to hit most of the balls sent by the defenders without bouncing, thus decreasing the frequency of the off the wall smash, using safer smashes like trays. However, the greater continuity of the hits for a good defense after the smash makes it necessary for the attacking players to look for other types of tactical actions that allow them to provoke successful situations in the point. Thus, it seems that varying the directions of the shots has been one of the fundamental tactical principles to achieve success in racket sports [25,36]. The results of this study showed that both men and women vary the directions of the down the line and cross court smashes in a balanced way, which would produce more movement on the part of the opponents swinging from one side of the court to the other, which may imply that they hit in more unfavorable situations making more mistakes [12,13].

Gender differences showed that men made a significantly higher percentage of winners than women (10% more topspin smashes and almost 20% more flat smashes). These results could be due to the anthropometric and strength differences between elite men and women players [37,38]. The results of these studies show that men padel players are taller, with greater muscle percentage and higher levels of vertical jump and grip strength than the women players, which would allow them to use the powerful smash successfully in positions further from the net. Regarding the hitting directions, it was observed that the women performed a significantly higher percentage of cross court flat and topspin smashes than the men. These differences may have a tactical explanation. While the men finish off a powerful smash down the line aiming to bring the ball to their field after the bounce on the back wall, the women do the cross court smashes with the aim of getting the ball to go over the side wall of the court (3 m high) [20]. In this way, the down the line smash, if not done with a lot of power, can cause the ball to bounce off the back wall at the opposite end with less force, offering a very favorable position for the return by the defending players [29]. With the results, coaches should work mostly on the flat stroke for the men and women, although women have a lower chance of winning than men.

In line with our hypothesis, males and females use one type of spike more than another, and with different performance (mainly in flat and top-spine strokes). The results of this study have important practical applications for the training of padel players, facilitating the design of tasks and exercises, as well as preparing them for competition taking into account the differences between the men’s and women’s categories. Moreover, knowledge of the effectiveness of the different types of smash depending on the court area in which the player is located will allow the training of perceptual and decisional mechanisms during the game by the player and the application of feedback about the behaviors by the coach [39,40]. However, this study has certain limitations that need to be taken into account when interpreting the results. For example, the use of some contextual variables such as the score (winning, drawing or losing) or the importance of the point (key moment) can influence decision-making in moments of pressure (choking) affecting performance [41]. In addition, other variables that influence smash effectiveness such as the position of the opponents or the speed of the smash have not been evaluated [29].

## 5. Conclusions

The tray was the most commonly used smash by padel players. Female players used more tray and less flat and topspin smashes than male players. Tray represents a percentage of point continuity of almost 90%. The flat and topspin smashes were the shots that achieved the highest percentage of winners, although this efficiency decreased significantly when the players moved away from the net area, especially in the flat smash. Regarding gender, men performed a significantly higher percentage of winning smashes than women. In addition, with regard to direction, flat smashes and off the wall smashes were predominantly down the line strokes and women performed significantly more cross court topspin smashes than men.

## Figures and Tables

**Table 1:** Efficacy of the stroke depending on the type of smash and the gender of the players.

Type of Smash	Efficiency	Gender				
		Male		Female		Sig.
		*N*	%	*N*	%	
Tray	Continuity	261	89.7	295	87.0	0.189
	Winner	21	7.2	23	6.8	
	Error	9	3.1	21	6.2	
Flat	Continuity	61	43.9a	53	63.1b	0.020 *
	Winner	72	51.8a	28	33.3b	
	Error	6	4.3	3	3.6	
Topspin	Continuity	29	56.9	15	62.5	0.305
	Winner	21	41.2	7	29.2	
	Error	1	2.0	2	8.3	
Off the wall	Continuity	31	75.6	32	69.6	0.514
	Winner	7	17.1	7	15.2	
	Error	3	7.3	7	15.2	

Note: *N* = Number; % = Percentage; * = *p* < 0.05; a, b = indicate significant differences in the Z tests for comparison of column proportions from *p* < 0.05 adjusted according to Bonferroni.

**Table 2:** Hitting direction depending on type of smash and players’ gender.

Type of Smash	Direction	Gender				
		Male		Female		Sig.
		*N*	%	*N*	%	
Tray	Down the line	151	51.89	169	49.85	0.610
	Cross court	140	48.11	170	50.15	
Flat	Down the line	108	77.70	57	67.86	0.105
	Cross court	31	22.30	27	32.14	
Topspin	Down the line	25	49.02	4	16.67	0.007 *
	Cross court	26	50.98	20	83.33	
Off the wall	Down the line	26	63.41	31	67.39	0.697
	Cross court	15	36.59	15	32.61	

Note: *N* = Number; % = Percentage; * = *p* < 0.05
